# An Analysis of the Binding Characteristics of a Panel of Recently Selected ICAM-1 Binding *Plasmodium falciparum* Patient Isolates

**DOI:** 10.1371/journal.pone.0111518

**Published:** 2014-10-31

**Authors:** Aymen M. Madkhali, Mohammed O. Alkurbi, Tadge Szestak, Anja Bengtsson, Pradeep R. Patil, Yang Wu, Saeed Alharthi, Anja T. R. Jensen, Richard Pleass, Alister G. Craig

**Affiliations:** 1 Department of Parasitology, Liverpool School of Tropical Medicine, Liverpool, United Kingdom; 2 Department of Medical Laboratories Technology, College of Applied Medical Sciences, Jazan University, Jazan, Kingdom of Saudi Arabia; 3 Department of Laboratory Medicine, College of Applied Medical Sciences, Umm Al-Qura University, Makkah Al-Mukarramah, Kingdom of Saudi Arabia; 4 Centre for Medical Parasitology, Department of International Health, Immunology and Microbiology, Faculty of Health and Medical Sciences, University of Copenhagen, Copenhagen, Denmark; Bernhard Nocht Institute for Tropical Medicine, Germany

## Abstract

The basis of severe malaria pathogenesis in part includes sequestration of *Plasmodium falciparum*-infected erythrocytes (IE) from the peripheral circulation. This phenomenon is mediated by the interaction between several endothelial receptors and one of the main parasite-derived variant antigens (PfEMP1) expressed on the surface of the infected erythrocyte membrane. One of the commonly used host receptors is ICAM-1, and it has been suggested that ICAM-1 has a role in cerebral malaria pathology, although the evidence to support this is not conclusive. The current study examined the cytoadherence patterns of lab-adapted patient isolates after selecting on ICAM-1. We investigated the binding phenotypes using variant ICAM-1 proteins including ICAM-1^Ref^, ICAM-1^Kilifi^, ICAM-1^S22/A^, ICAM-1^L42/A^ and ICAM-1^L44/A^ using static assays. The study also examined ICAM-1 blocking by four anti-ICAM-1 monoclonal antibodies (mAb) under static conditions. We also characterised the binding phenotypes using Human Dermal Microvascular Endothelial Cells (HDMEC) under flow conditions. The results show that different isolates have variant-specific binding phenotypes under both static and flow conditions, extending our previous observations that this variation might be due to variable contact residues on ICAM-1 being used by different parasite PfEMP1 variants.

## Introduction

Malaria is still a life-threatening disease despite the huge effort to eliminate it. WHO estimated about 207 million cases of malaria, and 627 000 fatalities reported in 2012, with most of these occurring in African children under five years old [1]. Malaria severe disease syndromes such as cerebral malaria (CM) are thought to involve the adhesion between the parasite derived antigens and a subset of host receptors [2].

The mature stages of the asexual intraerythrocytic cycle of *Plasmodium falciparum* are not found in the circulation due to their ability to localise to different organs in a phenomenon known as sequestration. Understanding some of the key events that facilitate sequestration is important to identify possible targets to develop either inhibitors or vaccines [3]. *Plasmodium falciparum* erythrocyte membrane protein-1 (PfEMP-1) [4]–[6] mediates sequestration with various human receptors [3] including ICAM-1 [7] and CD36 [8], [9] and EPCR [10].

One of the commonly used receptors, ICAM-1, has a cytoplasmic tail, a transmembrane domain and five extracellular Ig-like domains [11], [12]. The main function of ICAM-1 on endothelial cells is to allow leukocyte transmigration from the blood to the tissues in inflammatory sites upon cytokine stimulation. ICAM-1 binds to leukocyte receptors such as leukocyte function-associated antigen (LFA-1) or macrophage-1 antigen (Mac-1). In addition, ICAM-1 mediates binding to pathogens, such as human rhinoviruses (HRVs) [12], and *P. falciparum* infected erythrocytes [7]. The ICAM-1 binding sites for IE, rhinoviruses, LFA-1 and fibrinogen are overlapping, but also have distinct regions [13], [14].

Several lines of evidence support the involvement of ICAM-1 in malaria pathogenesis. First, a study carried out on post-mortem samples taken from people diagnosed with CM showed accumulation of IE in brain vessels that co-localised with ICAM-1 [15]. In addition, ICAM-1 was found up-regulated in brain accompanied with *P. falciparum* infection [16]. Isolates from severe malaria patients and particularly from CM demonstrated higher binding to ICAM-1 [10], [17], [18], although this is not seen in all studies. Another line of evidence is the association between CM and a homozygous mutation in ICAM-1 in Kilifi, Kenya, named ICAM-1^Kilifi^ [19], although it should be noted that other studies such as those in the Gambia and Thailand have not shown an association between ICAM-1^Kilifi^ and severe malaria [20], [21] [12]. In contrast, ICAM-1^Kilifi^ was suggested to have a protective role in Gabon [22]. Previous studies have characterised ICAM-1 binding phenotypes under both static and flow conditions on purified proteins and endothelial cells. These have shown that IE have subtle differences in binding to ICAM-1 with different affinities and avidities ranging from 2.8 nM to 144 nM for a number of PfEMP1 variants from the IT lineage [23].

CD36 is an integral membrane protein expressed on various host cells including endothelium, and platelets [3]. CD36 is implicated in the regulation of membrane transport systems, immune responses in humans [24], [25] and platelet adhesion [26]. CD36 is a common receptor for almost all *P. falciparum* isolates in field studies, albeit with some notable exceptions. Adhesion to CD36 is seen for IE from severe or uncomplicated malaria patients [17], [27] except isolates from pregnancy-associated malaria [28], [29], although in some studies it has been associated with uncomplicated malaria isolates [9]. Also, CD36 adhesion protects from CM in South East Asia [30]. Recently, it has been suggested that CD36 may protect against anaemia caused by malaria [31].

PfEMP-1 proteins are encoded by 50–60 extremely variable *var* genes per parasite genome [32]. Despite this variation of the *var* genes, they can be grouped into three major groups; A, B, and C based on their promoter sequence and chromosomal locations [33], [34]. Group A *var* genes are less diverse than the others and have been found to be associated with severe malaria [35]. A typical PfEMP-1 structure consists of two to seven Duffy-binding–like (DBL) domains and one to two cysteine-rich interdomain region (CIDR) domains [32]. Specific domains have been implicated in binding to certain host receptors [36]. A recent sub-classification for DBL and CIDR domains from seven genomes has led to the identification of shared combinations of short tandem domain cassettes (DCs) in many different parasite strains [37]. Among the Group A PfEMP-1 s, there are several ICAM-1– binding DBL domains isolates identified so far, including PFD1235w, Dd2var32 [35] and PF11_0521 [38], [39]. Furthermore, from these isolates, it was possible to identify a novel tandem three-domain PfEMP1 region called DC4, and antibodies to this region have been found to be cross-reactive with group A PfEMP1 proteins that bind to ICAM-1 [40]. However, although DC8 and DC13 cassettes found in Group A PfEMP1 proteins were associated with SM [41]–[43], IE expressing DC8 and DC13 were found not to bind to ICAM-1 [41], [43].

In the current study, we have investigated the binding phenotypes for ICAM-1-selected, recently lab-adapted patient isolates under static and flow adhesion assays. We analysed the role of four mutant ICAM-1 variants that have previously shown different effects on laboratory isolates [44] and the effect of four anti-ICAM-1 mAb using static assays, expanding the number of the isolates in comparison with previous studies. Understanding key events in cytoadherence is important in identifying possible targets in order to develop either effective inhibitors or vaccines.

## Methods

### Parasite culture

Laboratory lines, ItG [45] and A4 [14] and lab-adapted patient isolates PO-69, 8206, 8146, 8131, 6392 (from Kenya) J1, PCM-7, BC-12 and GL-6 (from Thailand [46]) were cultured at 1% haematocrit in O+ human erythrocytes using standard culturing techniques, [47] using complete medium (RPMI 1640 medium supplemented with 37.5 mM HEPES, 7 mM D-glucose, 6 mM NaOH, 25 mg/ml of gentamicin sulphate, 2 mM L-glutamine, and 10% human serum) at a pH of 7.2 in a gas mixture of 96% nitrogen, 3% carbon dioxide, and 1% oxygen. A batch of parasite stabilates selected on recombinant ICAM-1 was made to minimise the effect of mixed populations and antigenic switching. The parasites were used in binding assays for only three weeks post-selection.

All patient isolates were collected with consent as part of clinical studies in Kenya and Thailand, and all patient material has been removed during culture, replaced with blood sourced commercially from the UK Blood Transfusion Service.

### ICAM-1 Selection

50 µl Protein A Dynabeads (Invitrogen) were washed 3 times with 200 µl 1% BSA/PBS using a magnet to retain the beads each time, and then resuspended in 200 µl PBS/1%BSA. 2.5 µg/ml ICAM-1^Ref^ protein was added to the bead suspension. The mixture was rotated at 15 rpm at room temperature for 60 minutes. ICAM-1 labelled Dynabeads were purified on the magnet, washed three times in 1% BSA/PBS, and resuspended in 200 µl 1% BSA/PBS. Parasite culture was enriched for mature stages using Plasmion by standard protocols [12]. The enriched IE were incubated with ICAM-1 labelled Dynabeads and rotated for 45 minutes at room temperature. Unbound parasites were removed by three gentle washes with 1% BSA/PBS. IE-bound beads were resuspended in complete media with fresh washed red blood cells and cultured as standard.

### Parasite genotyping

Genotyping was carried out to ensure the distinctiveness of each isolate in this study and that cross contamination of parasite cultures had not occurred. The primers and reactions used were based on the Recommended Genotyping Procedures (RGPs) to identify parasite populations (Medicines for Malaria Venture, Amsterdam May 2007). The protocol was accessed through http://www.who.int/malaria/publications/atoz/rgptext_sti.pdf. DNA was extracted at trophozoite stage then purified using QIAamp DNA Blood Mini Kit (QIAGEN).

### Recombinant proteins

ICAM-1-Fc reference (ICAM-1^Ref^ [48]), ICAM-1 Kilifi (ICAM-1^Kilifi^), ICAM-1 S22/A (ICAM-1^S22/A^), ICAM-1 L42/A (ICAM-1^L42/A^) and ICAM-1 L44/A (ICAM-1^L44/A^) [44] have been described previously.

### Static adhesion assays

Purified recombinant proteins were spotted in triplicate in a radial pattern using 2 µl spots on 60 mm plastic petri dishes (Falcon 1007; Becton Dickinson, Oxford, UK) at concentrations of 50 µg/ml for ICAM-1, two plates were prepared for each parasite isolate. The dishes were incubated in a humidified chamber for 2 hours at 37°C to allow the proteins to adsorb to the surface, after that the spots were aspirated off and the plastic petri-dishes were filled with 1% BSA/PBS, blocking buffer, and incubated overnight at 4°C. The plates were warmed at 37°C for one hour prior the assay. IE were suspended in binding buffer (RPMI 1640 R4130 (Sigma, Dorset, UK) in 2% glucose at pH 7.2) at 3% parasitemia and 1% haematocrit. The blocking buffer was removed from the dish prior to adding 1.25 ml of the IE suspension. The plates were incubated at 37°C for one hour with gentle resuspension every 10 minutes. Then, the IE suspension was removed by gentle manual washing (4–6 washes) with binding buffer medium. The bound IE were fixed with 1% glutaraldehyde in PBS for 1 hour and stained with 10% Giemsa for 20 minutes. Six pictures were captured for each spot under 900x magnification using software HC Image (Sewickley, USA) to give 18 readings per plate for each isolate and in total 36 readings from the two plates. The pictures were analysed by Image-Pro version 7 (Rockville MD, USA). The means of at least three independent experiments performed on different days were calculated using Microsoft Excel 2010. The results were expressed as the mean number of IE bound per mm^2^ of surface area or, when appropriate, as a percentage of the control binding for the mutant ICAM-1s and mAbs inhibition data.

### Static inhibition assays

The same static binding technique described above was applied with the addition of mAbs at 5 µg/ml to the IE suspension prior adding it to the plates. All the mAbs were commercially available; 15.2 (AbD serotec), My13 (Invitrogen), 8.4A6 (Sigma), BBIG-I1 (R&D systems).

### Flow assay

#### HDMEC culture

HDMEC cells were obtained from Promocell (C-12210). HDMEC media (Promocell C-39220, 500 ml) was supplemented with EC growth media (Promocell C-39215).

Sub-culturing followed the standard protocol following manufacturer’s instructions. Cells were washed with HEPES-buffered Balanced Salt Solution), trypsinised and neutralised. However, for the flow assay Accutase (Sigma) was used as an alternative detaching reagent instead of trypsin.

#### VenaEC preparation

Details about the system can be found on the Cellix website via: http://www.cellixltd.com/. TheVenaFlux is a semi-automated microfluidic system able to perform cell adhesion studies under shear flow mimicking *in* *vivo* flow rates. It is designed to facilitate the study of cell adhesion, and to be more physiologically relevant than static assays. Its construction makes it easier to use than previous systems used to mimic physiological rates of flow in vessels for *P. falciparum* adhesion studies. The system includes VenaEC 8-channels designed for growth of human endothelial cells with continuous feeding during the experiment and parameters that can be adjusted and monitored during the experiment through the VenaFlux software.

VenaEC 8-channells (Cellix - Dublin, Ireland) were coated with 12 µl of 100 µg/ml fibronectin and incubated in a humidified petri-dish at 4°C overnight. Cells were activated with 10 ng/ml TNF 16–24 hours before the day of the assay. On the following day, VenaEC 8-channels were warmed at 37°C for 30 minutes. The endothelial cells (EC) were treated with Accutase, detached and then neutralised with medium. The EC were pelleted at 300×*g* for 3 minutes and resuspended in an appropriate volume EC medium to achieve 1.5×10^6^ cells/ml. Then, 5 µl of this suspension were seeded onto each channel and incubated at 37°C. Once the cells attached to the channels, they were fed every 30 minutes until the EC become confluent, usually within 2–3 hours. The IE suspension for both binding and inhibition assays was prepared as described in static assays except the haematocrit was adjusted to 2%.

The assay was run following the Cellix protocol using the VenaFlux software. VenaEC 8-channels were connected to Cellix system in a microscope stage enclosed within a plastic chamber to keep the temperature at 37°C. The flow through the channels was adjusted to run 0.04 Pa and the IE suspension was drawn through the channel for five minutes. After that, binding buffer was passed through the cell at the same rate to wash for two minutes. The bound IE were counted in six fields and converted to the number of IE/mm^2^. For binding inhibition, all mAbs were used at 5 µg/ml. The IVC7 anti-CD36 mAb was kindly provided by Prof. Ellen van der Schoot (Sanquin, Amsterdam).

### Ethics Statement

The parasites were derived from two main sources. The collection of the Kenyan patient isolates and use in adhesion assays was approved by the Research Ethics Committees at KEMRI, Kenya and the Liverpool School of Tropical Medicine [18]. The Thai isolates were originally derived from patients from the Thai-Cambodia and Thai-Myanmar borders and were supplied for this study from Thammasat University as laboratory adapted isolates [46].

## Results

### Binding to ICAM-1^Ref^

**Figure 1:**
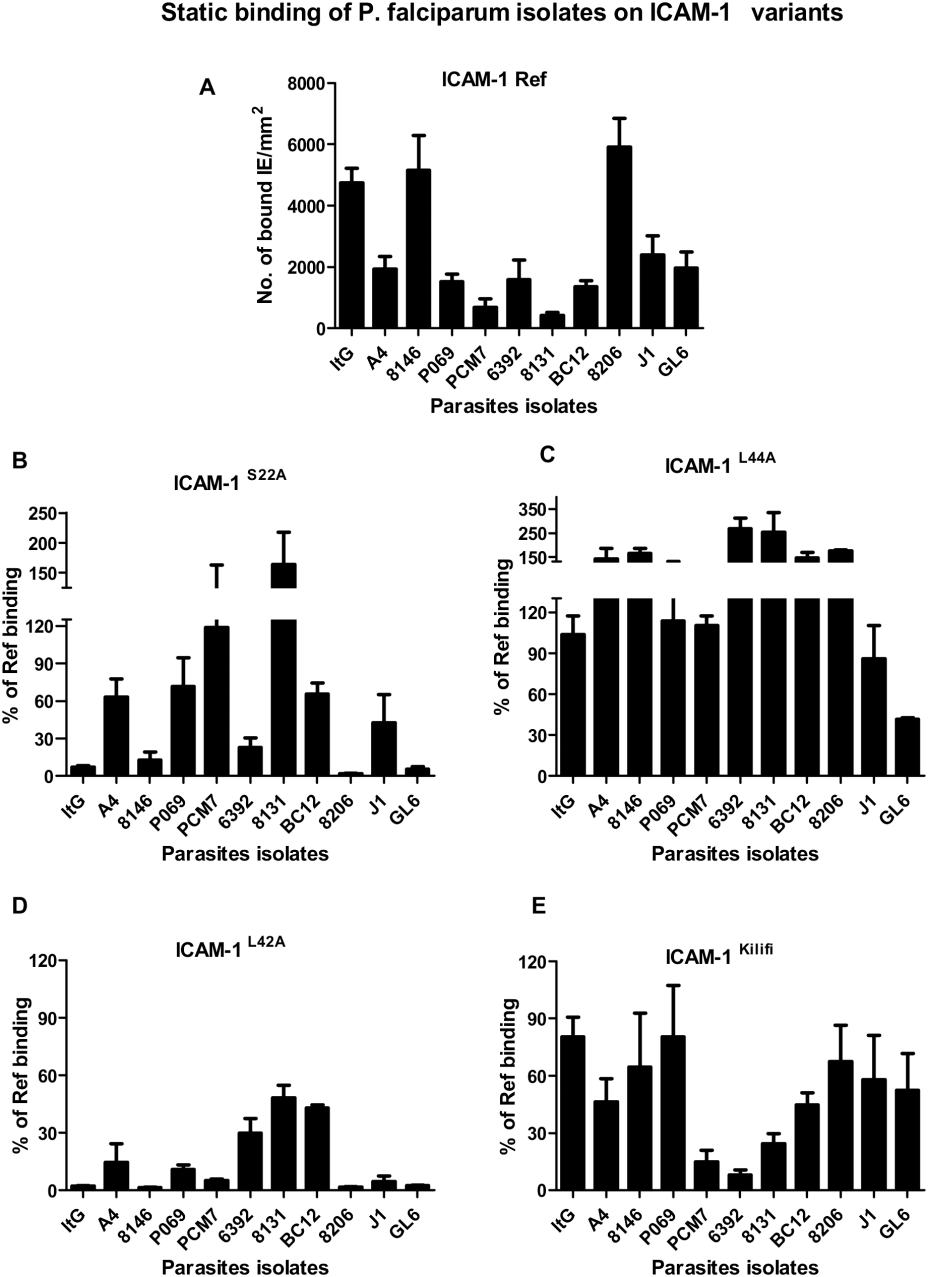
Static adhesion assay, 2 µl spots at 50 µg/ml protein concentration were placed onto 6 cm dishes and standard protein static binding assays carried out with IE suspended in binding buffer at a parasitaemia of 3% and a haematocrit of 1%. The results show the mean of binding (IE/mm^2^) (Figure 1A) and %ICAM-1^Ref^ binding (Figure 1B–E), and the bars represents SE (n≥3).

All isolates were genetically distinct as shown by genotyping (data not shown). Based on the level of binding to ICAM-1^Ref^, all the isolates were categorised into high and low-avidity parasites. ItG was previously defined as a high-avidity ICAM-1 binder whereas, A4 was characterised as low-avidity ICAM-1 binder from an earlier study [49]. Using the same criteria only two of the lab-adapted isolates were high-avidity ICAM-1 binders; 8146 and 8206. The rest of the isolates were assigned as low-avidity binders (Figure 1A).

### Binding to mutant ICAM-1 proteins

The current study showed that there were considerable differences in IE binding to mutant ICAM-1 proteins (Figure 1). The S22/A mutation severely reduced the binding of the high-avidity group and some of the low-avidity isolates; 6392, and GL6 by about 80%. In addition, there was a moderate effect on PO69, BC12 and J1 (Figure 1B). On the other hand, L42/A mutation showed a critical effect on all of the isolates, reducing binding by at least 50% compared to ICAM-1^Ref^, with the binding almost totally inhibited for most of isolates, including the high-avidity group (Figure 1C). By contrast, L44/A mutation reduced the binding of GL6 only, and increased the binding for some isolates (Figure 1D). The binding of isolates was variably disturbed by the ICAM-1^Kilifi^ polymorphism. ICAM-1^Kilifi^ affected the binding of four isolates by about 50%. Moreover, there were three isolates was reduced by more than 75%, PCM7, 6392 and 8131. Whereas, the ItG and PO69 isolates was only reduced by 20%. Statistical tests comparing low and high avidity isolates did not show a significant difference between these populations, although this was highly affected by the small number (n = 3) of high avidity isolates available (Figure 1E).

### ICAM-1 Static Inhibition Assays

**Figure 2:**
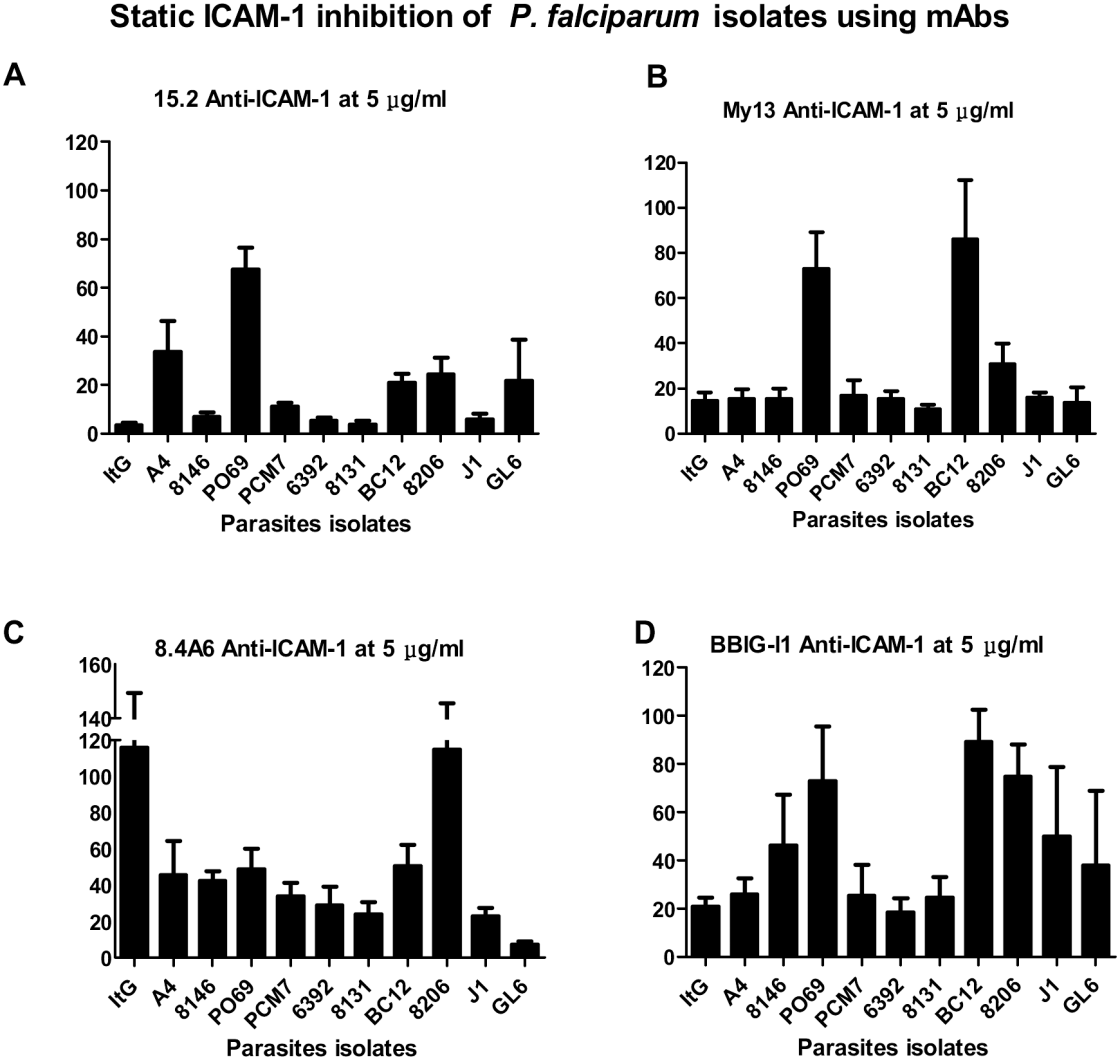
Static adhesion assay. The same method as for Fig. 1 but with the addition of 5 µg/ml ofmAbs 15.2 (A), My13 (B), 8.46A (C) and BBIG-I1 (D) prior the IE incubation with ICAM-1^Ref^. The results show the percentage of binding in the presence of ICAM-1 mAb compared to the no mAb treatment (A), and the bars represents SE (n>3).

The effect of anti-ICAM-1 mAbs on the binding of IE to purified ICAM-1 under static conditions has been studied using specific mAbs reacting with epitopes on Ig-like domains 1 and 2. MAbs 15.2, BBIG-I1 and My13 mapping to domain 1, and 8.4A6 mAb mapping to domain 2 were used in a study that differentiated between the binding sites on ICAM-1 for IE and LFA-1 [13]. Figure 2 (and Data S2) show that different mAbs have different inhibitory effects on the isolates. The results are expressed as the percentage of the binding of each isolate against its binding to ICAM-1^Ref^. The binding of most of the isolates was reduced by about 75% by two mAbs 15.2 and My13. However, there was only 40% inhibition caused by 15.2 and My13 to PO69 (Figure 2A–B). The inhibition caused by 8.4A6 was different. The effect varied between 25–50% for most of the isolates, although there was no effect by 8.4A6 on the ItG and 8206 isolates (Figure 2C). The range of inhibition for BBIG-I1 was between 25%–75% for nearly all isolates except there was almost no effect on 8206 (Figure 2D). These variations again suggest the use of variable contact residues between ICAM-1 and variant PfEMP-1 proteins.

### Flow endothelial adhesion assay

**Figure 3:**
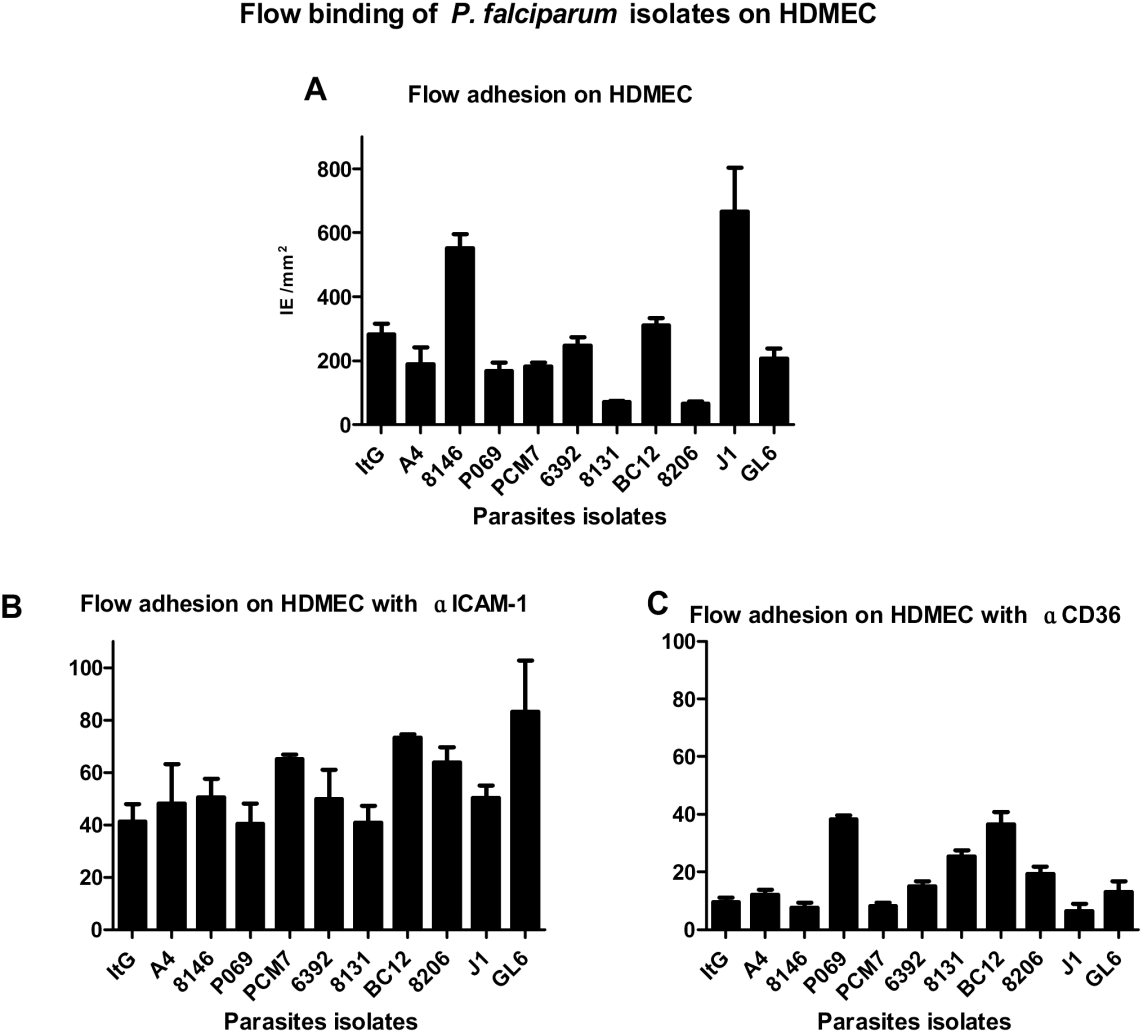
Flow endothelial cell adhesion assay. HDMEC seeded on channels pre-coated with fibronictin; IE were passed on confluent cells for five minutes followed by washing by binding buffer for two minutes before counting 6 fields in two different channels. The parasitaemia was 3% and a haematocrit of 2%. The results show the mean of binding with no inhibitory mAbs (A) and the % binding on treatment with 5 µg/ml of 15.2 anti-ICAM-1 (B) and 5 µg/ml IV-C7 anti-CD36 (C) mAbs compared to no mAb treatment. The bars represents SE (n≥3).

The binding level on HDMEC under flow conditions is similar for seven out of eleven isolates tested within the range 200–300 IE/mm^2^ (Figure 3 and Data S3). In contrast, two isolates, 8146 and J1, bound within the range 600–700 IE/mm^2^ to HDMEC despite J1 being assigned as low-avidity ICAM-1 binder on purified ICAM-1. Another two isolates, 8206 and 8131, showed relatively less binding to HDMEC although 8206 was categorised as a high-avidity ICAM-1 binder on purified ICAM-1. Further investigation using an anti-ICAM-1 mAb revealed similar activity for 15.2 mAb on almost all isolates, reducing binding by approximately 50% (Figure 3B). However, the binding was reduced by about 80% for eight isolates in the presence of the anti-CD36 mAb and for the other three was reduced by about 60% (Figure 3C).

## Discussion

The aim of this study was to establish the binding characteristics of a set of new ICAM-1 binding isolates to provide further information about the interaction between ICAM-1 and PfEMP-1. The purpose of using field isolates is usually to investigate the association between binding phenotypes and clinical outcomes. The selection of ICAM-1 binding PfEMP-1 populations in this study introduces bias by potentially expanding small sub-populations from the original sample and so cannot be used to derive associations between the clinical outcomes and binding phenotypes. Our original study used three genetically distinct ICAM-1-binding laboratory isolates (two of which (ItG and A4) are included in this work for comparison), screened against 25 mutant ICAM-1 proteins using static and flow adhesion systems [44].

Based on this previous work, binding and inhibition assays were run on a larger number of recently lab-adapted isolates using the ICAM-1 mutations previously shown to disrupt the binding and discriminating between laboratory isolates, using static assays only. Binding to endothelial cells was investigated using a flow adhesion system.

Alanine replacement mutagenesis and ICAM-1-specific mAbs have given more details about the binding region on ICAM-1 for *P. falciparum*-infected erythrocytes. The binding between IE and ICAM-1 was revealed to involve the BED face of ICAM-1, including the DE loop [44]. The binding phenotypes from previous studies were categorised based on the isolate’s avidity to ICAM-1. Overall, the new binding and inhibition data support the original findings that different ICAM-1-binding isolates can use different contact residues in the DE loop of ICAM-1 to bind. Moreover, current data support previous findings by demonstrating a significant role for L42 for all ICAM-1-binding isolates.

Two ICAM-1-specific mAbs My13 and 15.2, which have been mapped to epitopes including the L42 residue, reduced the binding of all the isolates. The binding of low-avidity-ICAM-1 isolates was more affected by these mAbs than high-avidity-ICAM-1 parasites. 8.4A6 ICAM-1 mAb, which targets an epitope on domain 2, can also inhibit the binding of all isolates. This might be explained by the epitope in domain 2 being in a position close to domain 1 or affecting the structure of this domain, as they have been shown to interact to produce the native ICAM-1 structure [13]. Interestingly, most of the isolates were low-avidity ICAM-1 binders similar to A4, which was previously associated with a signature that reflects isolates from SM cases [18].

Flow adhesion on endothelial cell assays more accurately resembles the situation seen in the human circulation than static assays [12]. In the present study, we used TNF-activated HDMEC, which expresses both ICAM-1 and CD36 receptors as well as other endothelial receptors, using the Cellix system to measure IE adhesion. The Cellix system has produced comparable results with earlier flow-based systems on various endothelial cells (data not shown). The binding to activated HDMEC was approximately the same level for most (7/11) isolates, comparable to ItG and A4, which bind in the range 200–300 IE/mm^2^. The binding was reduced with both anti-ICAM-1 and anti-CD36 mAbs with the latter causing greater inhibition than anti-ICAM-1. Binding to CD36 is shown in Data S1. This might be explained by ‘receptor co-operation’ between ICAM-1 and other receptors [12], [50]. It is likely that ICAM-1 is not the only receptor involved in CM pathogenesis and, for example, a recent study has associated the ability of IE to bind to EPCR with severe malaria, including CM [10], [51].

ICAM-1 has been suggested to play a capturing role from the circulation thereby contributing to the pathogenesis of CM [49]. However, the role of CD36 in sequestration is not understood. CD36 binding is a characteristic phenotype for the majority of paediatric isolates and in some studies has been shown to be more associated with adhesion to isolates from UM cases. It has been suggested that the host utilises CD36 to control the parasitemia prior to host immune responses or to minimise pro-inflammatory responses [18].

The molecular basis of the variable binding to ICAM-1 is thought to be due to differences in the contact residues between this receptor and the variant PfEMP1s. PfEMP-1 binds to ICAM-1 through a diverse set of DBLβ domains mainly from groups B or C and it would be difficult to target DBLβ domains in these groups due to their extensive sequence diversity. This is seen particularly in approaches to discriminate ICAM-1 binding DBLβ domains from non-binding ones, which has only been partially successful [52]. There are ICAM-1 binders among the group A PfEMP-1 that contain a definable DC4 cassette [40], but this is still at a very preliminary stage and needs more investigation to see if it could provide a starting point for the development of a vaccine targeting CM by inhibiting IE sequestration via ICAM-1 in the brain. The variability in the binding characteristics between IE and ICAM-1 suggests that it could be a difficult problem to find a cross-blocking therapy, although the central role of the L42 residue and anti-DC4 blocking antibodies provides some support for this approach.

The divergent binding pattern to variants of ICAM-1 of different IE is similar to that demonstrated by the causative agent of the common cold, Human Rhinovirus (HRV). The major serotypes of HRV utilise ICAM-1 to invade the epithelium and two different HRV serotypes have shown varying adhesion phenotypes to ICAM-1^Ref^ and ICAM-1^Kilifi^, and their association with varying clinical outcome [53]. Very recently, an anti-human ICAM-1 antibody that specifically binds domain 1 of human ICAM-1, prevented entry of two major groups of rhinoviruses, reduced virus burden, cellular, inflammation and pro-inflammatory cytokine induction *in* *vivo*. Importantly, this antibody did not affect ICAM-1 binding to LFA-1, leaving this critical host pathway intact [54]. Similar approaches could be used to lead to the development of novel treatments candidates to reduce malaria morbidity and mortality but require a good understanding of the variety of IE adhesion to ICAM-1.

The key outcome of this study is the identification of vital targets on the sites of the interaction between parasite ligands and host receptors, which may lead to the development of inhibitors which target IE sequestration. Despite the presence of rapid and effective parasite-killing drugs, deaths still occur among children with severe malaria complications, particularly in the immediate period after admission to hospital. Several strategies to improve survival in malaria have been highlighted in a recent review [55]. One of these is targeting parasite adhesion to the vascular endothelium. Anti-adhesion therapeutics is an encouraging project in the discovery of novel treatments, including compounds based on the structure of endothelial receptors [56]. High-throughput screening could identify adhesion blocking molecules that inhibit IE from binding or activating microvascular endothelium [55]. An example of this type of rational-inhibitor design is (+)-EGCG, a polyphenol compound showing significant inhibition ranging from 37%–80% by the new ICAM-1-binding isolates used in the present study [57]. The variation of inhibition by (+)-EGCG might be due to the variable contact residues on PfEMP-1 of different patient isolates. The mode of action of (+)-EGCG is assumed to be its structural simulation of part of the ICAM 1 binding site for IE based around the L42 loop.

In conclusion, the isolates tested use variable contact residues on ICAM-1 for their binding. However, L42/A inhibits all isolates binding, supporting the idea of a conserved region on ICAM-1 for PfEMP-1 binding and that could be used to target interventions.

## Supporting Information

Data S1 **Adhesion data of** ***P. falciparum*** **isolates on ICAM-1 variants under static adhesion assays.** Numbers represent the means of adherent IE/mm^2^ in independent experiments and the % compared to ICAM-1^Ref^ binding for the mutant ICAM-1 proteins. Data for binding to CD36, where available, is also provided (means and SD, NA: no data available).
(XLSX)

Data S2 **Inhibition data of** ***P. falciparum*** **isolates under static inhibition assays.** Data represents the means of the binding in the presence and absence of mAbs in independent experiments and the % of the binding in the presence of mAbs compared to no mAb binding. It should be noted that the numbers expressed here are/field and not/mm^2^.
(XLSX)

Data S3 **Adhesion data of** ***P. falciparum*** **isolates to HDMEC under flow adhesion assays.** Data represents the means of independent experiments/mm^2^ and their standard deviation and number of experiments for each isolate.
(XLSX)
