# Correction: An Analysis of the Binding Characteristics of a Panel of Recently Selected ICAM-1 Binding *Plasmodium falciparum* Patient Isolates

**DOI:** 10.1371/journal.pone.0148836

**Published:** 2016-02-05

**Authors:** Aymen M. Madkhali, Mohammed O. Alkurbi, Tadge Szestak, Anja Bengtsson, Pradeep R. Patil, Yang Wu, Saeed Alharthi, Anja T. R. Jensen, Richard Pleass, Alister G. Craig

The seventh author’s name is spelled incorrectly. The correct name is: Saeed A. Al-Harthi.

The affiliation for the seventh author is incorrect and the correct affiliation is not indicated. Saeed A. Al-Harthi is not affiliated with #3. The correct affiliation is: Department of Parasitology, Faculty of Medicine, Umm AL-Qura University, Makkah, Kingdom of Saudia Arabia.
